# Transforming growth factor-β1 downregulates tryptophanyl-tRNA synthetase expression in human lung fibroblasts

**DOI:** 10.1016/j.advms.2026.02.002

**Published:** 2026-02-16

**Authors:** Aaron K. McDowell-Sanchez, Konstantin Tsoyi

**Affiliations:** a Department of Medicine, Section of Pulmonary, Critical Care, and Sleep Medicine, Baylor College of Medicine, Houston, United States; b Department of Medicine, Division of Pulmonary and Critical Care, University of Maryland Baltimore, Baltimore, United States

**Keywords:** Pulmonary fibrosis, Lung fibroblasts, WARS1, Transforming growth factor-beta

## Abstract

**Purpose:**

The role of tryptophanyl-tRNA synthetase, also known as WARS, in lung fibroblasts is currently un-known. We aimed to study the effect of transforming growth factor-beta 1 (TGF-β1) on the expression of WARS and whether it regulates profibrotic responses in TGF-β1-activated human lung fibroblasts.

**Materials and methods:**

We used MRC-5, a human lung fibroblast cell line, and primary human lung fibroblasts (HLFs) derived from control subjects. WARS expression was measured by enzyme-linked immunosorbent assay (ELISA) and Western blot. Profibrotic responses in TGF-β1-stimulated human lung fibroblasts were measured by Western blot, gel contraction assay, and real-time quantitative PCR (RT-qPCR).

**Results:**

We demonstrate that TGF-β1 potently downregulates WARS expression, both at the extracellular and intracellular levels, in MRC-5, and HLFs. Yin Yang 1 (YY1) transcription factor (TF) silencing ameliorates the inhibitory effect of TGF-β1 on WARS expression. Finally, we found that recombinant (r) WARS significantly inhibited fibronectin (FN) but had no effect on collagen1 (COL1) or alpha-smooth muscle actin (αSMA) expression in TGF-β1-induced HLFs.

**Conclusions:**

Our results demonstrate that TGF-β1 inhibits WARS expression via YY1, however, WARS treatment has a modest effect on regulating profibrotic responses in activated HLFs.

## 1. Introduction

Idiopathic pulmonary fibrosis (IPF) is a serious chronic lung disorder of undefined etiology [1]. Fibroblasts are considered one of the main effector cells in the pathogenesis of organ fibrosis, including IPF [2]. Fibroblasts are known to undergo myofibroblast transformation, produce excessive extracellular matrix (ECM) proteins, and initiate contractile protein activation in response to profibrotic factors such as transforming growth factor-beta (TGF-β). The result of dysregulated fibroblast profibrotic activity is tissue remodeling and fibrosis [3].

Fibroblasts may also contribute to the progression of pulmonary fibrosis by releasing proteins that promote inflammation and activation of other cells in the lung. For example, the expression of interleukin-6 (IL-6), CCL2, also known as monocyte chemoattractant protein-1 (MCP-1), IL-11, or connective tissue growth factor (CTGF) is elevated in activated lung fibroblasts, which are known as profibrotic and proinflammatory mediators [4]. However, the full portfolio of expressed cytokines in human lung fibroblasts (HLFs) in profibrotic conditions is not fully elucidated.

Tryptophanyl-tRNA synthetase/WARS is an essential housekeeping enzyme that promotes the addition of tryptophan into the amino acid chain during the translation of mRNA [5]. Besides its canonical function, WARS can be secreted in response to cellular stress and serve as a proinflammatory cytokine [6,7]. Indeed, WARS has been shown to exert monocyte/macrophage activation during bacterial and viral infection as well as angiogenesis in endothelial cells [6,8,9]. Here, we found that WARS expression is significantly downregulated in HLFs after TGF-β1 stimulation, but not in response to other profibrotic factors such as PDGF-BB, IL-6, adenosine triphosphate (ATP), and IL-11. Accordingly, the TGF-β type I receptor (TβRI) blockade completely reversed the TGF-β1-mediated inhibitory effect on WARS expression. Next, we observed that Yin Yang 1 (YY1) silencing recovered WARS expression in TGF-β1-stimulated lung fibroblasts suggesting that YY1 transcription factor plays an important role in TGF-β1-mediated WARS downregulation. Finally, we found that WARS treatment inhibited TGF-β1-induced fibronectin (FN) expression but did not regulate collagen1 (COL1) or alpha-smooth muscle actin (αSMA) levels nor fibroblast contractility.

Taken together, our results suggest that TGF-β1-stimulation potently inhibits WARS expression in HLFs. However, exogenous WARS treatment has a modest effect on fibroblast activation in response to TGF-β1.

## 2. Materials and Methods

### 2.1. Reagents

Recombinant human WARS was obtained from Novus Biological (Cat#: NBP1-50843; Centennial, CO, USA). Human IL-6 and IL-11 were obtained from PeproTech (Cat#: 200-06-100UG and 200-11-100UG, respectively; Cranbury, NJ, USA). ATP was acquired from Sigma-Aldrich (Cat#: A7699; St. Louis, MO, USA). TGF-β1 was obtained from Sino Biological (Cat. # 10804-HNAC; Beijing, China). Rapalink-1 was obtained from Cell Signaling Technology (Cat#: 88626; Danvers, MA, USA). eCF506, SB431542, wortmannin, LY3214996, SP600125, and SB202190 were obtained from Cayman Chemical (Cat#: 19959, 13031, 10010591, 27936, 10010466, and 10010399, respectively; Ann Arbor, MI, USA). All other reagents were acquired from Sigma-Aldrich.

### 2.2. Cells

HLF cell line (MRC-5) cells were obtained from ATCC (Cat #: CCL-171; Manassas, VA, USA). Deidentified primary HLFs from control subjects were obtained under an Institutional Review Board (IRB)-approved protocol (Protocol number: H-50814) at the Baylor College of Medicine (Houston, TX, USA). Cells were periodically tested for myco-plasma contamination using a commercially available kit from ATCC (Cat#: 30–1012K).

### 2.3. Enzyme-linked immunosorbent assay (ELISA)

Measurements of WARS in culture medium were performed by a commercially available human WARS ELISA Kit (Cat# MBS703294) following instructions provided with the kit from MyBioSource (San Diego, CA, USA).

### 2.4. Lentiviral transfection

Empty plasmid (pBMN) and a plasmid carrying artificial microRNA (amiRNA) precursor and shRNA to silence human YY1 (pBMN-AS-YY1) were obtained from Addgene (Cat#: 80389 and 154943 respectively; Watertown, MA, USA). Lentiviral particles were generated by co-transfecting HEK293T cells with target plasmid (pBMN or pBMN-AS-YY1) and 3rd Generation Packaging Mix (Cat#: LV053; Applied Biological Materials, Richmond, Canada) according to manufacturer’s instructions. Then, MRC-5 cells were treated with lentiviral particles, 5 multiplicity of infection (MOI). Stably transfected cells were selected using puromycin (10 μg/mL) from Sigma-Aldrich.

### 2.5. Western blot

Polyacrylamide gel electrophoresis and immunoblotting were performed according to standard methods. Briefly, protein concentration was measured using a BCA protein assay kit (Cat#: 23227) from Thermo Fisher Scientific (Waltham, MA, USA). Equal amounts of protein were mixed with pre-stained loading buffer and boiled for 5 min 20 μg of the total protein per sample were electrophoresed on the precast polyacrylamide gels (Cat#: 4569036) provided from Bio-Rad Laboratories (Hercules, CA, USA). Then, the electrophoresed proteins were transferred to polyvinylidene difluoride (PVDF) membranes (Cat#: 03010040001; MilliporeSigma, Burlington, MA, USA) by wet electrophoretic transfer at 100 V for 60 min. The PVDF membranes were blocked for 1 h at room temperature in 5% bovine serum albumin (BSA) and then incubated with primary antibody at 4 °C overnight. Antibodies to αSMA, COL1, FN and YY1 were obtained from Abcam (Cat#: ab5694, ab21286, ab2413, and ab109237, respectively; Cambridge, MA, USA); β-actin from Santa Cruz Biotechnology (Cat#: sc-47778, Santa Cruz, CA, USA); WARS from Novus Biologicals (Cat#: NBP2–32186). Next day, membranes were washed in Tris Buffered Saline-Tween-20 (TBST) buffer and incubated for 1 h with horseradish peroxide (HRP)-conjugated anti-rabbit or anti-mouse secondary antibodies from Santa Cruz Biotechnology (Cat #: sc-2357 and sc-516102, respectively). Signals were detected using Chemiluminescent Substrate kit (Cat#: 34580) from Thermo Fisher Scientific. Images were taken using ChemiDoc imaging system (Cat#: 12018025) from Bio-Rad Laboratories. Quantification of protein bands was performed with the computer software ImageJ and was expressed as a ratio of fold change band intensity with respect to the loading control [10]. Quantification of immunoreactive protein bands was performed with the computer software ImageJ and was expressed as a ratio of fold change band intensity with respect to the loading control.

### 2.6. Cell contraction assay

Cell pellet was reconstituted in 1 × PBS and mixed with rat collagen I solution with final collagen concentration of 1 mg/mL 0.5 mL of collagen solution containing 1 × 10^6^ cells was poured into 24-well plate and incubated for 1 h at 37 °C to allow gelation. Then, gels were released from plate with spatula and 1 mL of culture media was added. Gel’s size was measured by the ruler 24 h after TGF-β1 treatment.

### 2.7. Chromatin immunoprecipitation (ChIP) assay

The ChIP assay was performed using an enzymatic Chromatin IP kit from Cell Signaling Technology (Cat#: 9002S), according to the manufacturer’s instructions. In brief, MRC-5 cells were lentivirally transfected with pBMN or pBMN-AS-YY1 as described above. Then, cells were exposed to TGF-β1 (10 ng/mL) or not for 24 h and then fixed in 1% formaldehyde for 10 min at room temperature. Cross-linking was stopped by adding glycine. DNA was digested by the use of micrococcal nuclease to a length of ~150–900 bp. Before incubation with antibodies, 10 μL of input control solution was taken from each sample. The remaining chromatin solution was incubated with 10 μg anti-YY1 antibody at 4 °C overnight (Santa Cruz Biotechnology)). Immune complexes were precipitated, washed, and eluted. DNA-protein cross-links were reversed by heating at 65 °C for 2 h, and 10 μL of each sample was used as a template for qRT-PCR. The WARS1 oligonucleotide sequences for PCR primers were: forward primer, 5′-TGT AAT CCC AGC TAC TCG GGA-3′, and reverse primer, 5′-AGT AAA AAA TTA CCA AAG CAC-3′. The primer set encompasses the WARS1 promoter segment from nucleotide 671 to 530.

### 2.8. Statistical analysis

Data are expressed as mean ± standard error the mean (SEM). For comparisons between the two groups, we used Student’s unpaired *t*-test. One-way analysis of variance followed by Newman-Keuls or Tukey’s post-test analysis was used for the analysis of more than two groups. The numbers of samples per group (n), or the number of experiments, are specified in the figure legends. Statistical significance was defined as p < 0.05.

### 2.9. Ethical issues

Primary control HLFs were obtained from deidentified subjects. The study was approved by the Institutional Review Board of Baylor College of Medicine, which waived the requirement for formal consent. All procedures were conducted in accordance with the 1964 Declaration of Helsinki with its later amendments.

## 3. Results

### 3.1. TGF-β1 downregulates WARS expression in MRC-5 cells

**Fig. 1.**
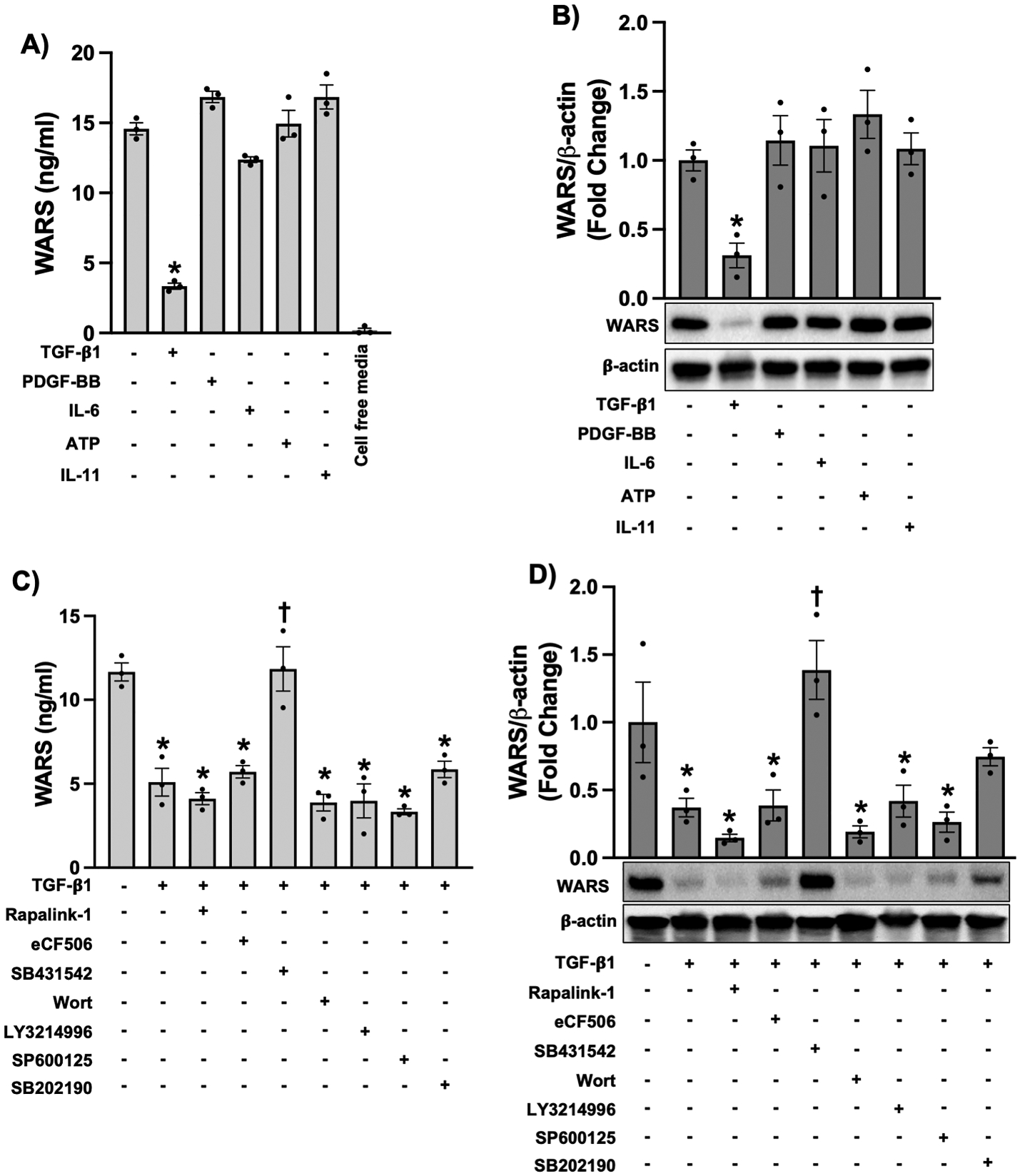
TGF-β1 downregulates WARS expression in MRC-5 cells. **A** and **B)** MRC-5 cells were stimulated with TGF-β1 (10 ng/ml), PDGF-BB (10 ng/ml), IL-6 (10 ng/ml), ATP (1 mM) or IL-11 (20 ng/ml). 24 h later, culture media samples were harvested and subjected to ELISA to measure WARS (A), and cells were lysed to conduct a western blot to measure intracellular levels of WARS (B) (n = 3). **C** and **D)** MRC-5 cells were stimulated with TGF-β1 (10 ng/ml) in the presence or absence of Rapalink-1 (10 nM), eCF506 (10 μM), SB431542 (10 μM), wortmannin (100 nM), LY3214996 (10 μM), SP600125 (10 μM), or SB202190 (10 μM). 24 h later, culture media samples were harvested and subjected to ELISA to measure WARS (C), cells were lysed to conduct western blot to measure intracellular levels of WARS (D) (n = 3). Data are mean ± SEM. P < 0.05; significant comparisons by one-way ANOVA: * vs. unstimulated, † vs. TGF-β1 alone.

MRC-5 cells were stimulated with TGF-β1 (10 ng/mL), PDGF-BB (10 ng/mL), IL-6 (10 ng/mL), ATP (1 mM), or IL-11 (20 ng/mL) for 24 h. Only TGF-β1 stimulation induced downregulation of WARS expression levels at both extracellular and intracellular levels in MRC-5 cells (Fig. 1A and B). Other cytokines had no statistically significant effect on WARS expression (Fig. 1A and B). As expected, TGF-β type I receptor inhibition with SB431542 (10 μM) could fully restore WARS expression after TGF-β1 treatment (Fig. 1C and D). Interestingly, the mammalian target of rapamycin complex-1 (mTORC1), Src, phosphoinositide 3-kinase (PI3K), and mitogen-activated protein kinase (MAPK) signaling inhibitors did not statistically significantly affect the WARS level in TGF-β1-stimulated MRC-5 cells (Fig. 1C and D).

### 3.2. YY1 regulates WARS expression in TGF-β1-stimulated MRC-5 cells

**Fig. 2.**
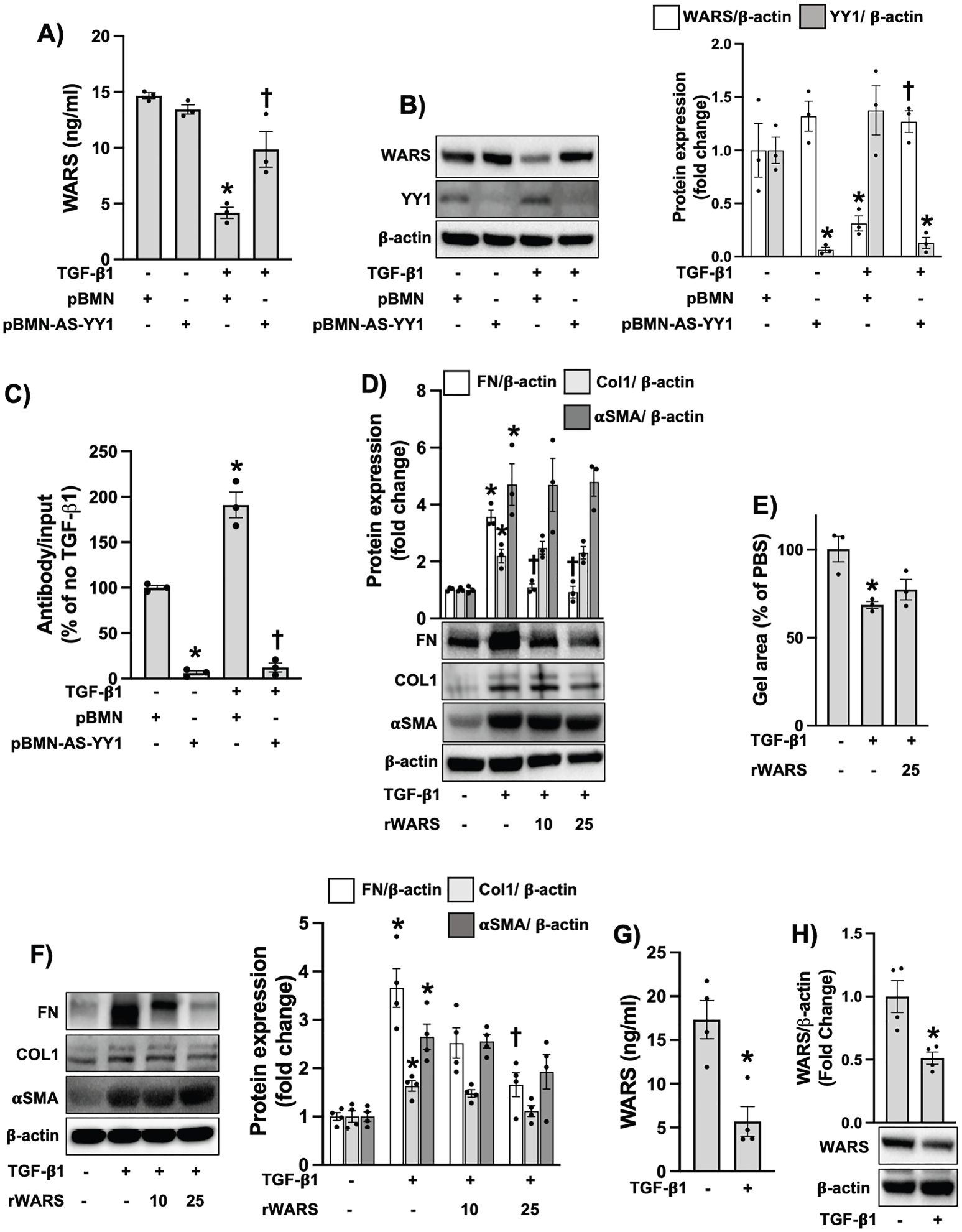
WARS is regulated by YY1 and ameliorates fibronectin expression in activated human lung fibroblasts. **A** and **B)** MRC-5 cells were lentivirally transfected with empty vector (pBMN) or pBMN-AS-YY1 as described in Methods. Then, stably transfected cells were selected with puromycin 10 (μg/ml) for 7 days. After selection, cells were stimulated with TGF-β1 (10 ng/ml) for 24 h. WARS levels were measured by ELISA (A) and western blot (B) (n = 3). **C)** MRC5 cells were lentivirally transfected with pBMN or pBMN-AS-YY1 and treated with TGF-β1 as described above. After TGF-β1 treatment cells were fixed with 1% formaldehyde harvested and subjected to ChIP assay as described in Methods (n = 3). **D)** MRC-5 cells were stimulated with TGF-β1 (10 ng/ml) in the presence or absence of recombinant (r) WARS (10 ng/ml, 25 ng/ml). The treatment of TGF-β1 and WARS has been performed simultaneously. 24 h later, cells were lysed for Western blot to measure Fibronectin, Type I Collagen, and αSMA expression (n = 3). **E**) MRC-5 cells were gelated as described in Methods. Then, gels were treated with TGF-β1 (10 ng/ml) in the presence or absence of rWARS (25 ng/ml). 24 h later gel size was measured by the ruler (n = 3). **F**) Primary human lung fibroblasts (HLFs) from control subjects were treated with TGF-β1 (10 ng/ml) in the presence or absence of rWARS (10 ng/ml, 25 ng/ml). 24 h later, cells were lysed for western blot to measure Fibronectin, Type I Collagen, and αSMA expression (n = 4). **G** and **H**) HLFs were stimulated with TGF-β1 (10 ng/ml) for 24 h. Then, culture media and cell lysate were collected to measure WARS by ELISA (F) and western blot (G) respectively n = 4. Data are mean ± SEM. P < 0.05; significant comparisons by one-way ANOVA or Student t-test: * vs. unstimulated, † vs. TGF-β1 alone.

Using short hairpin (sh) and small interference (si) RNA techniques to silence YY1, we tested whether TGF-β1-mediated inhibitory effect on WARS expression is dependent on YY1 expression. As shown in Fig. 2A and B, and Supplementary Figure S1 B, we found that silencing YY1 restores the WARS expression in MRC-5 cells after TGF-β1 stimulation. Finally, using ChIP assay, we found that TGF-β1 increases YY1 binding to its recognition site GCCAT on WARS1 promoter (Fig. 2C). Taken together, our data suggest that WARS expression is dependent on YY1 transcriptional activity but not on mTORC1, Src, PI3K, or MAPK signaling in activated lung fibroblasts.

### 3.3. WARS attenuates FN expression in TGF-β1-activated human lung fibroblasts

Since the WARS level is downregulated after TGF-β1 stimulation, we asked whether rWARS treatment reverses the profibrotic responses in activated lung fibroblasts. As shown in Fig. 2D and E and F, we could observe that WARS treatment statistically significantly inhibited FN expression but had no effect on αSMA and COL1 levels or cell contractility in TGF-β1-stimulated MRC-5 and primary HLFs. We did not observe that rWARS alone impacted the activation of lung fibroblasts (Supplementary Figure S1 C). Of note, we also observed a statistically significant downregulation in WARS expression in primary HLFs after TGF-β1 stimulation (Fig. 2G and H).

## 4. Discussion

Cell injury and inflammation are important triggers of organ fibrosis [11]. Thus, multiple cytokines and chemokines are known to be involved in the development of both inflammation and fibrosis.

WARS, a recently identified cytokine, is normally localized in the cytosol conducting the ligation of tryptophan to its cognate tRNA for protein synthesis [5]. However, under hyperinflammatory conditions, such as sepsis, its extracellular level is increased and acts as a cytokine by promoting inflammation, which may ultimately lead to organ failure and death via TLR4 activation [6,8]. In endothelial cells, WARS has been shown to regulate angiogenesis. In particular, the alternative splice form of WARS, termed mini-WARS, potently inhibited angiogenesis by interacting with VE-cadherin [12,13]. Also, it has been shown that N-terminally truncated fragment of WARS inhibited extracellular signal-regulated kinase 1/2 (ERK1/2) and Akt signaling pathways in endothelial cells under the shear stress [14]. These prior reports strongly suggest that WARS plays a major role in various biological processes such as inflammation and blood vessel formation beyond its classical function as an enzyme which catalyzes the addition of tryptophan into the amino acid chain.

Recently, another tRNA-synthetase, prolyl-tRNA synthetase 1 (PARS1), has been shown to be involved in the development of pulmonary fibrosis [15]. It has been shown that PARS1 deficiency and inhibition ameliorate pulmonary fibrosis by interfering with the synthesis of ECM proteins, such as collagens which are known to have a high content of PARS1-dependent amino acids, like proline. However, the role of WARS in fibroblasts is not fully understood. We found that WARS level was statistically significantly downregulated in TGF-β1-stimulated human lung fibroblasts. Previously, it has been published that the YY1 transcription factor plays an important role in profibrotic responses in TGF-β1-activated lung fibroblasts [16]. Further, it has been suggested that YY1 may serve as both a transcription activator and suppressor of genes in activated fibroblasts [16,17]. We found that silencing YY1 transcription factor recovered WARS expression in TGF-β1-stimulated lung fibroblasts suggesting that TGF-β1-mediated effect of WARS expression is regulated by YY1.

Treatment cells with rWARS downregulated FN expression but did not have any effect on COL1 or αSMA in TGF-β1-stimulated cells. We also found a mild downregulation of IL-6 in WARS-treated lung fibroblasts induced by TGF-β1 (Supplementary Figure 1 D). At the same time, the expression of CCL2, IL-11, or CTGF was not affected by WARS (Supplementary Figure 1 D). The molecular mechanism by which WARS treatment may ameliorate FN and IL-6 expression in lung fibroblasts remains enigmatic. However, it is possible that WARS may mediate its effects by interacting with TLR4 [6], cadherin-6 [18] or by regulating tryptophan levels in activated lung fibroblasts [19]. Future studies could be focused on identifying these mechanisms and the effect of WARS on other cell types during the progression of pulmonary fibrosis.

### 4.1. Limitations of the study

In this study, we focused on understanding the role of WARS in lung fibroblasts. The limitation of this study is that we did not study the role of WARS in other cell types of the lung, such as alveolar epithelial cell and macrophages, which are known to play a significant role in pulmonary fibrosis.

## 5. Conclusions

Here, we found that WARS levels are significantly suppressed after TGF-β1 stimulation in HLFs. Administration of WARS ameliorated FN and IL-6 expression but did not affect other profibrotic proteins and cytokines in TGF-β1-activated HLFs, suggesting that rWARS has a modest effect on lung fibroblast activation.

## Supplementary Material

1

## Data Availability

The data that support the findings of this study are available from the corresponding author upon reasonable request.

## Appendix A. Supplementary data

Supplementary data to this article can be found online at https://doi.org/10.1016/j.advms.2026.02.002.
