# Supplementary material for: Transforming growth factor-β1 downregulates tryptophanyl-tRNA synthetase expression in human lung fibroblasts
Source: Adv Med Sci. Author manuscript; Available in PMC 2026 May 27. (PMC13215394; doi:10.1016/j.advms.2026.02.002)
Supplement: 1 [file NIHMS2178249-supplement-1.docx]

1. **Supplementary Methods**

*1.1 mRNA isolation and qRT-PCR*

Total RNA was isolated with RNeasy Universal Kit (Qiagen, Valencia, CA, USA) and cDNA was synthesized from RNA (1 μg) using a SuperScript First-strand synthesis system for reverse transcription (RT) PCR (Invitrogen, Carlsbad, CA, USA). RT-PCR, with SYBR Green Master Mix (Bio-Rad Laboratories, Hercules, CA, USA), was performed using the StepOnePlus Real-Time PCR System (Applied Biosystems, Waltham, MA, USA). The relative quantity of target mRNA was calculated using the CT method, or 2–∆∆CT, as described by Schmittgen et al. [1], and normalized using glyceraldehyde-3-phosphate dehydrogenase (GAPDH) as an endogenous control (Sequence Detection System software, version 1.7; Applied Biosystems). Primer sequences are presented in Supplementary Table S1.

*1.2 Transient Transfection*

Scramble (Scr) or siYY1 were obtained from Santa Cruz Biotechnology (Cat#: sc-37007 and sc-36863, respectively). Transient transfections were performed using FuGENE 6 (Promega, Madison, WI, USA) in accordance with the manufacturer’s instructions.

**References**

[1] Schmittgen TD, Livak KJ. Analyzing real-time PCR data by the comparative C(T) method. Nat Protoc. 2008;3:1101-8.

**Supplementary Figure S1. Effect of WARS on cytokine expression in MRC-5 cells.** **A)** MRC-5 cells were transiently transfected with Scr or siYY1 and incubated overnight. Next day, cells were lysed and transfection efficiency was assessed by Western blot (n=3). **B)** Scr or siYY1 transfected MRC-5 cells were stimulated with TGF-β1 (10 ng/mL) or not. 24 hours later, culture media samples were harvested and subjected to ELISA to measure WARS (n=3). **C)** MRC-5 cells were incubated with WARS (10 and 25 ng/mL) or TGF-β1 (10 ng/mL) for 24 hours. Then, cells were lysed to conduct a Western blot to measure intracellular levels of FN (n=3). **D)** MRC-5 cells were stimulated with TGF-β1 (10 ng/mL) with or without WARS (25 ng/mL). 24 hours later, culture media samples were harvested and subjected to RNA isolation and qRT-PCR as described in Supplemental Methods (n=3). Data are presented as mean ± SEM. P < 0.05; significant comparisons by one-way ANOVA: * vs. unstimulated, † vs. TGF-β1 alone or Scr+ TGF-β1.


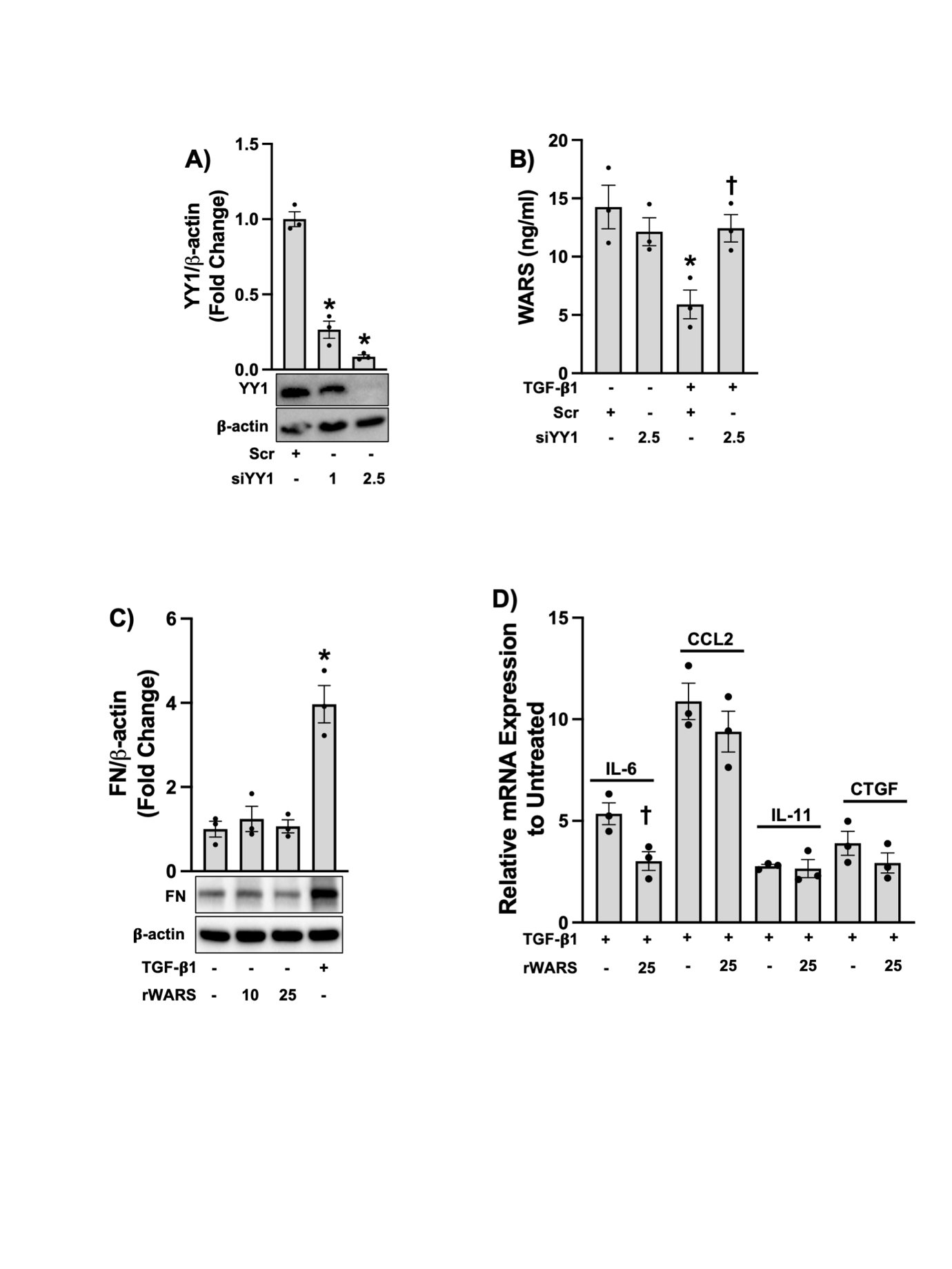


**Supplementary Table S1. Table of primers used in measuring mRNA expression of target genes.**

| Gene | Forward | Reverse |
| --- | --- | --- |
| human IL-6 | 5′-GGTACATCCTCGACGGCATCT-3′ | 5′-GTGCCTCTTTGCTGCTTTCAC-3′ |
| human CCL2 | 5′-TCGCGAGCTATAGAAGAATCA-3′ | 5′-TGTTCAAGTCTTCGGAGTTTG-3′ |
| human IL-11 | 5′-GGACCACAACCTGGATTCCCTG-3′ | 5′-AGTAGGTCCGCTCGCAGCCTT-3′ |
| human CTGF | 5′-GTTTGGCCCAGACCCAACT-3′ | 5′-GGAACAGGCGCTCCACTCT-3′ |
| human GAPDH | 5′-GGATTTGGTCGTATTGGG-3′ | 5′-GGAAGATGGTGATGGGATT-3′ |
